# Computer-associated health complaints and sources of ergonomic instructions in computer-related issues among Finnish adolescents: A cross-sectional study

**DOI:** 10.1186/1471-2458-10-11

**Published:** 2010-01-11

**Authors:** Paula T Hakala, Lea A Saarni, Ritva L Ketola, Erja T Rahkola, Jouko J Salminen, Arja H Rimpelä

**Affiliations:** 1 Tampere School of Public Health, FIN-33014 University of Tampere, Tampere, Finland; 2 Tampere University Hospital, P.O Box 2000, FIN-33421, Tampere, Finland; 3 City of Helsinki, Health Center, P.O Box 6100, FIN-00099, Helsinki, Finland; 4 Finnish Institute of Occupational Health, Topeliuksenkatu 41 a A, 00250 Helsinki, Finland; 5 Rovaniemi University of Applied Sciences, Porokatu 35, 96400 Rovaniemi, Finland; 6 Department of Physical and Rehabilitation Medicine, University Hospital of Turku, P.O.Box 52, FIN-20520 Turku, Finland

## Abstract

**Background:**

The use of computers has increased among adolescents, as have musculoskeletal symptoms. There is evidence that these symptoms can be reduced through an ergonomics approach and through education. The purpose of this study was to examine where adolescents had received ergonomic instructions related to computer use, and whether receiving these instructions was associated with a reduced prevalence of computer-associated health complaints.

**Methods:**

Mailed survey with nationally representative sample of 12 to 18-year-old Finns in 2001 (n = 7292, response rate 70%). In total, 6961 youths reported using a computer. We tested the associations of computer use time and received ergonomic instructions (predictor variables) with computer-associated health complaints (outcome variables) using logistic regression analysis.

**Results:**

To prevent computer-associated complaints, 61.2% reported having been instructed to arrange their desk/chair/screen in the right position, 71.5% to take rest breaks. The older age group (16-18 years) reported receiving instructions or being self-instructed more often than the 12- to 14-year-olds (*p *< 0.001). Among both age groups the sources of instructions included school (33.1%), family (28.6%), self (self-instructed) (12.5%), ICT-related (8.6%), friends (1.5%) and health professionals (0.8%). Receiving instructions was not related to lower prevalence of computer-associated health complaints.

**Conclusions:**

This report shows that ergonomic instructions on how to prevent computer-related musculoskeletal problems fail to reach a substantial number of children. Furthermore, the reported sources of instructions vary greatly in terms of reliability.

## Background

Pain symptoms in the neck and lower back increased among Finnish adolescents in the 1990s and this trend has continued in the 2000s [1]. At the same time there has been a remarkable increase in the use of computers [2], as in the risk of several musculoskeletal health complaints [3-5].

Pain symptoms have been measured using separate questions, for e.g. headache [3,4], neck-shoulder pain [3,5] and low back pain [5-7], which have been observed as more common among computer users than among non-users. A dose-response relationship between computer use time and the occurrence of symptoms has been noted in a previous study [5]. Young computer users' own perceptions on whether computer use has induced these symptoms have also been investigated. Results have suggested that computer use is associated with neck-shoulder or back pain [8-14] but also pain in the hands, fingers or wrists [8,10,11,13,15] and eyes [8,10]. Most of the studies have been cross-sectional with unrepresentative, small samples that cannot be relied upon to draw conclusions on causality.

There is evidence that musculoskeletal symptoms can be reduced through an ergonomics approach and through education. In a study by Henning *et al*. [16], frequent short breaks from computer work reduced musculoskeletal discomfort and other computer-related complaints among adults. In a randomized trial, Ketola *et al*. [17] investigated the impact of an intensive ergonomics approach and education on workstation changes and musculoskeletal disorders among adult visual display unit users, and found that after two months, both the intensive ergonomics and the education groups had less musculoskeletal discomfort than the reference group. Among young people and adolescents, 6^th ^grade children who reported not having furniture specifically designed for computer use were more likely to have musculoskeletal discomfort than those who had [9]. Educational programmes with a focus on overall postural health, body mechanics and environmental ergonomics have enhanced the knowledge regarding computer habits among students, including a non-stressful body position while working at the computer [18,19].

Based on research and best practice evidence to avoid musculoskeletal complaints induced by computer work, professional bodies such as the Finnish Institute of Occupational Health http://www.ttl.fi advise appropriate ergonomics as well as sufficient resting breaks among adults. Computer users need to know how to create good ergonomic working arrangements for computer workstations, including placement of the screen, keyboard, mouse and lighting. Furthermore, they need to be aware of the importance of short breaks to decrease the stress in soft tissues, discs and nerves caused by the static postures and prolonged sitting frequently involved in computer use. Digital computers designed for the use of the general public are still newcomers to our society. The fact that their appropriate use, with respect to personal health, requires specific learning and instructions has not necessarily been recognized by either schools or parents. There is a lack of studies as to whether children receive instructions on how to prevent e.g. musculoskeletal problems, and whether receiving these instructions is related to a decreased prevalence of health complaints.

The primary aim of this study was to examine a large population sample of 12 to 18-year-old adolescents to find out where they had received ergonomic instructions regarding computer workstation set-up and work practices, and secondly whether receiving these instructions was associated with a reduced prevalence of computer-associated health complaints.

## Methods

### Subjects

We used data from the Adolescent Health and Lifestyle Survey of 2001, a nationwide monitoring system of Finnish adolescents conducted biennially since 1977. The survey sample was drawn from the Population Register Centre by selecting all 12-, 14-, 16- and 18-year-old Finns born on certain adjacent dates in July. The Adolescent Health and Lifestyle Survey has been approved by the Ethical Committee of the Department of Public Health of the University of Helsinki. Self-administered questionnaires were mailed in February and followed by two reminder letters and questionnaires to non-respondents. The sample size was 10 360 (7292 responded, response rate 70%). The number of respondents and response rates among boys by age were: 351 (72%) 12-year-olds, 1251 (66%) 14-year-olds, 892 (62%) 16-year-olds and 774 (53%) 18-year-olds. Among girls, the corresponding figures were: 452 (82%) 12-year-olds, 1485 (79%) 14-year-olds, 1138 (82%) 16-year-olds and 976 (76%) 18-year-olds.

### Computer-related health complaints

The symptoms caused by excessive computer use were elicited for five different anatomical sites as follows: "Using a computer may cause health complaints (pains, aches, discomfort). Have you experienced any of these when using a computer?" The anatomical locations were: a) neck or shoulders, b) hands, fingers, wrists, c) lower back, d) head and e) eyes. The response alternatives for each item were a) not at all, b) sometimes and c) often. A summary variable was formed based on the number of locations where the respondents reported health complaints: a) none, b) 1, c) 2 to 3 or d) 4 to 5 anatomical sites. The reliability of different perceived symptoms in the previous surveys of the Adolescent Health and Lifestyle Survey have been tested and shown to be fair to good [20] when measured by kappa coefficients (0.47 for headache, 0.51 for abdominal pains, 0.42 for fatigue or weakness) [21].

### Computer use time

Computer use time was measured as follows: "How much time do you spend, on average, daily on a computer for e-mail, writing and searching for information?" The response alternatives in this structured question were: a) not at all, b) occasionally, c) <1 hour, d) 1-3 hours, e) 4-5 hours and f) >5 hours. A computer user was defined as an adolescent who used a computer at least occasionally (*N *= 6,961). Because of the small number of adolescents reporting computer use of over 4 hours daily, categories e) and f) were merged for analysis. In our previous paper based on the 2003 survey, we showed the reliability of the questions on computer time was fair to good when measured with kappa coefficients (0.45 for weekly use of computers, 0.65 for weekly use of the internet, 0.45 for daily use of computers) [5].

### Sources of ergonomic instructions

The type of ergonomic instructions received related to computer use was elicited using the question: "Were you ever instructed, or did you instruct yourself on how to avoid these health complaints?" Two alternatives, with response options No/Yes, were given: 1) I was instructed to arrange the desk, chair and screen in the correct position and 2) I was instructed to take a rest break and do something else for a while. Those (*N *= 238; 3.0%) who did not answer the question at all were excluded from the analysis. We used one additional question: "What was the source of these instructions?" To this open-ended question the respondents had provided between one and three answers, which were categorized into seven variables describing the sources of instructions: *school *(e.g. lessons related to health education, ergonomics, automatic data processing, physical education or work safety), *family *(parent, guardian, sibling, grandparent, relative), *friends *(friends, mates), *myself *(self-instructed, instructed through experience, realization or understanding), *information and communication technology (ICT) related *(computer literature, ICT professionals, instant messaging and programmes, television, radio), *health care professionals *(public health nurse, physiotherapist, doctor, eye specialist, other health care professional), *other sources or cannot say *(hobby, course, job, apprenticeship).

### Statistical analysis

Data were analysed using SPSS for Windows (version 11.0.; SPSS Inc, Chicago, Illinois). Using logistic regression analysis, the computer-associated health complaint variables were dichotomised into "often" and "never or sometimes". In the first analysis the association between health complaints (outcome variables) and computer use time (predictor variable) was tested, adjusting for age, sex and parents' education (primary school, vocational school, comprehensive school, matriculation examination, college or university). The second analysis tested the associations between computer-associated health complaints (outcome variables) and received ergonomic instructions (predictor variables). After first examining each health complaint in the model adjusted for age and sex, computer use time was included. Age, sex and computer use time variables were considered potential confounders and treated as covariate, because the response alternatives were categorical and the scale has irregular intervals. We calculated the odds ratios and 95% confidence intervals (Cl), and used *P*-values to show the differences between the age and sex groups. Analysis of non-respondents was tested by dividing the data into three categories according to the return date of the questionnaire. The statistical differences between the three respondent groups were tested using the chi-square test.

## Results

### Prevalence of computer-related health complaints

Computer users reported symptoms in multiple body locations; eye discomfort was the most common, and lower back symptoms the least common (Table 1). The prevalence of health complaints increased with age, and girls reported more symptoms than boys. The differences between the age and sex groups were statistically significant (*p *< 0.001). Of all the respondents, 24.9% reported having no health complaints at all, and 15.0% reported having four or five symptoms occurring sometimes or often. Of the computer users reporting often occurring health complaints, 7.7% reported one symptom and 0.4% reported four or five.

**Table 1:** Percentages of adolescents that reported computer-associated health complaints often or sometimes, by anatomical site, sex and age among computer users.

	Eyes		Neck, shoulders		Head		Hands, fingers, wrist		Lower back	
	Often % (n*)	Sometimes % (n)	Often % (n)	Sometimes % (n)	Often % (n)	Sometimes % (n)	Often % (n)	Sometimes % (n)	Often % (n)	Sometimes % (n)
**Sex**										
Boys	3.6 (115)	37.8 (1210)	2.4 (78)	33.5 (1073)	2.2 (72)	26.6 (852)	1.7 (56)	22.8 (729)	1.3 (41)	16.1 (514)
Girls	9.1 (360)	48.8 (1925)	5.9 (234)	46.8 (1846)	5.8 (229)	38.1 (1505)	2.7 (106)	29.2 (1152)	2.0 (78)	19.9 (784)
**Age**										
12--14	5.3 (181)	42.5 (1455)	2.9 (98)	37.7 (1290)	3.4 (117)	32.7 (1119)	1.7 (58)	23.8 (815)	0.9 (32)	14.7 (503)
16--18	7.9 (294)	45.1 (1680)	5.7 (214)	43.7 (1629)	4.9 (184)	33.3 (1238)	2.8 (104)	28.6 (1066)	2.3 (87)	21.4 (795)

**Total**	6.6 (475)	43.9 (3135)	4.4 (312)	40.8 (2919)	4.2 (301)	33.0 (2357)	2.3 (162)	26.3 (1881)	1.7 (119)	18.2 (1298)

*n = Number of cases

### Association between computer use time and health complaints

Of those who reported using a computer only occasionally, 7.0% reported that symptoms often occurred in the eyes, 4.2% in the neck or shoulders, 4.1% in the head, 1.8% in the hands, fingers, or wrists and 1.6% in the lower back. Daily computer use of 4 hours or more increased frequent health complaints statistically significantly in all anatomical sites (hands, fingers and wrists 8%, lower back 5%, head 4%, eyes 4%, neck, shoulder 3%) compared to those who only used a computer occasionally; 1-3 hours increased health complaints significantly in the neck or shoulders and in the hands, fingers, or wrists; <1 hour did not increase health complaints (Table 2).

**Table 2:** Odds ratios (OR) and their 95% confidence interval (Cl) for frequent computer-associated health complaints by time (hours) spent daily on a computer, among 12- to 18-year-old computer users, adjusted for age, sex and parents' education.

Computer use time	Health complaints often									
	**Eyes**		**Neck, shoulders**		**Head**		**Hands, fingers, wrists**		**Lower back**	
	n**	**OR (95%Cl)**	**N**	**OR (95%Cl)**	**N**	**OR (95%Cl)**	**N**	**OR (95%Cl)**	**N**	**OR(95%Cl)**

Not daily	319 *	1.0	181	1.0	180	1.0	89	1.0	73	1.0
< 1 hour daily	57	0.8 (0.6-1.0)	44	1.1 (0.8-1.5)	43	1.0 (0.7-1.5)	19	1.0 (0.6-1.5)	13	0.8 (0.4-1.4)
1-3 hours daily	68	0.8 (0.6-1.0)	67	**1.5 (1.1-2.0)**	58	1.3 (0.9-1.7)	39	**1.6 (1.1-2.3)**	22	1.1 (0.7-1.7)
≥4 hours daily	16	**2.0 (1.1-3.5)**	10	**2.2 (1.1-4.2)**	12	**2.8 (1.5-5.1)**	13	**5.4 (2.9-10.1)**	6	**2.6 (1.1-6.1)**

* The reference category is indicated by an odds ratio (OR) of 1.0. Odds ratios are given in bold when they indicate a statistically significant difference from the odds of the reference category (Not daily) at 95% confidence level (CI).

** n = Number of cases

### Received ergonomic instructions

Of all computer users, 61.2% reported having received instructions or being self-instructed to arrange the desk, chair and screen into the right position, and 71.5% reported having been advised to take rest breaks (Table 3). The older age group (16-18 years) reported receiving instructions or being self-instructed more often than the 12- to 14-year-olds (*p *< 0.001). The proportion of girls was smaller in the group that reported arranging desk, chair and screen in the right position, and larger in the group taking rest breaks compared to boys (*p *< 0.001).

**Table 3:** Proportion (%) of 12- to 18-year-olds who reported having received instructions or being breaks in order to prevent computer-associated health complaints, by sex and age.

	To arrange desk, chair and screen in the right position % (n*)	To take rest breaks % (n)
**Sex**		
Boys	62.0 (1951)	70.3 (2212)
Girls	60.6 (2367)	72.5 (2833)
Total	61.2 (4318)	71.5 (5045)
**Age**		
12--14	54.0 (1819)	67.8 (2284)
16--18	67.8 (2499)	75.0 (2761)
Total	61.2 (4318)	71.5 (5045)

*n = Number of cases

### Sources of ergonomic instructions

Adolescents reported several sources of ergonomic instructions for computer workstation set-up and work practices. Of these, the most common source was school, reported by 33.1% of the respondents (Table 4), the prevalence being higher among girls and in the 16- to 18-year age group (*p *< 0.001). The second most common source was family (28.6%), which was more common among boys, and the prevalence of which decreased with age (*p *< 0.001). The third most common answer was 'myself' (12.5%); girls reported being self-instructed more often than boys, and the prevalence increased with age (*p *< 0.001). ICT-related sources were named by 8.6%, boys more often than girls, and the prevalence increased with age (*p *< 0.001). Friends (1.5%) and health care professionals (0.8%) were the least commonly reported sources.

**Table 4:** Reported sources of ergonomic instructions to prevent computer-associated health complaints.

Sex	School % (n**)	Family % (n)	Myself (self-instructed) % (n)	ICT-related* % (n)	Friends % (n)	Health care professionals % (n)	Other or not defined % (n)
Boys	27.4 (864)	29.7 (935)	11.0 (345)	9.2 (291)	1.3 (41)	0.7 (22)	2.0 (64)
Girls	37.7 (1474)	27.7 (1083)	13.8 (538)	8.1 (316)	1.6 (63)	0.8 (31)	3.0 (117)
**Age**							
12--14	27.4 (923)	36.2 (1221)	10.7 (362)	6.7 (225)	1.3 (43)	0.6 (21)	2.4 (82)
16--18	38.4 (1415)	21.6 (797)	14.1 (521)	10.4 (382)	1.7 (61)	0.9 (32)	2.7 (99)

**Total**	33.1 (2338)	28.6 (2018)	12.5 (883)	8.6 (607)	1.5 (104)	0.8 (53)	2.6 (181)

Percentage of adolescents reporting each source by sex and age.

One respondent may have reported one or more sources.

*ICT = Information and communication technology

**n = Number of cases

### Association between ergonomic instructions and occurrence of health complaints

Having received ergonomic instructions to arrange the desk, chair and screen into the right position was not statistically significantly related to computer-associated health complaints (Table 5). For those who reported having received instructions to take rest breaks computer-associated health complaints in the eyes were slightly (*p *= 0.032) more common than for those who did not report such instructions.

**Table 5:** Odds ratios (OR) and their 95% confidence interval (Cl) for frequent computer-associated health complaints among 12- to 18-year-old computer users who received ergonomic instructions, adjusted for age, sex and computer use time, by type of instructions.

Ergonomic instructions	Health complaints often									
	Eyes		Neck, shoulders		Head		Hands, fingers, wrists		Lower back	
	n*	OR (95%Cl)	N	OR (95%Cl)	N	OR (95%Cl)	n	OR (95%Cl)	N	OR (95%Cl)
**To arrange desk, chair and screen in the right position**	285	0.9 (0.7-1.1)	206	1.3 (1.0-1.7)	178	0.8 (0.7-1.1)	107	1.4 (0.9-2.1)	82	1.2 (0.8-1.9)
**To take rest breaks**	364	**1.3 (1.0-1.6)**	241	1.2 (0.9-1.6)	228	1.2 (0.9-1.6)	126	1.4 (0.9-2.0)	95	1.4 (0.9-2.3)

The reference category is those who did not receive instructions and their OR is 1.0.

Odds ratios (OR) are given in bold when they indicate a statistically significant difference from the odds of the reference category (not-instructed) at 95% confidence level (CI).

* n = Number of cases

### Analysis of non-respondents

The data were divided into three categories according to the return date of the questionnaire (original questionnaire *N *= 4988, first reminder *N *= 1840, second reminder *N *= 464). It was assumed that the later the person answered the more he or she resembled a non-respondent. The prevalence of computer-associated health complaints declined but none of them reached a statistical significance. The prevalence among the respondent groups were 46.1% to the original questionnaire, 44.2% to the first reminder and 39.5% to the second reminder for the neck or shoulders; 29.2%, 27.2%, 26.8% for hands, fingers and wrists; 19.6%, 20.3%, 20.6% for lower back; 37.7%, 36.5%, 33.7% for head, and 51.9%, 48.0%, 45.5% for eyes, respectively.

## Discussion

Computer-associated health complaints were common in this study based on a large, nationwide survey of 12-18-year-old adolescents. The most frequently reported symptoms were in the eyes, neck or shoulders. Using a computer daily for one hour or more was associated with increased health symptoms in the upper extremities (neck or shoulders, hands, fingers and wrists), and the use of four hours or more was related to symptoms in all anatomical sites including the eyes, the head and the lower back. Approximately two thirds of the adolescents reported having received ergonomic instructions to arrange their work desk, chair and screen into the right position or to take rest breaks in order to prevent computer-associated health complaints. There were several sources of instructions, school and family being the most common, followed by self-instruction and ICT-related sources (e.g. the Internet). After adjusting for age, sex and computer use time, which influence the relationship between computer use and symptomatology, we did not find a statistically significant association between prior receipt of ergonomic instructions and decreased levels of reported symptoms.

In the current study, health complaints were more frequent among girls than boys and the prevalence increased with age. Our results are parallel with previous findings concerning sex or age differences [3,5,7,12,14,15].

Our study confirmed the results of previous, smaller studies in a large nationwide survey which found that the occurrence of physical discomforts in locations such as the eyes and the neck-shoulders were related to computer use [8,10]. Some previous studies have also shown that the risk for symptoms rises with increasing computer use exposure time for neck-shoulder pain [5], and overall musculoskeletal pain or discomfort [9,22]. This relationship was likewise confirmed in our current study with respect to symptoms in the neck or shoulder region and the upper extremities.

This study is the first to examine whether adolescents had received instructions in computer-related ergonomic issues, and look into the sources of these instructions, on the basis of self-reports. Nearly 40% of the respondents reported not having received instructions or being self-instructed on how to arrange the work desk, chair and screen into the right position, and nearly 30% on how to take rest breaks during computer use. Considering that today's children and adolescents in all age groups are frequent computer users, they could be expected to be familiar with these two ergonomic aspects and capable of applying them to their everyday life. Our findings indicate that a remarkable proportion of adolescents are not aware of computer-related ergonomic instructions, even if in survey questions like ours there could be a memory bias, decreasing the proportion of those who reported receiving instructions.

Based on the existing research evidence, the source of ergonomic instructions for computer work can be considered important from the point of view of the quality of such instructions. Although school emerged as the primary source of reported instructions in slightly over 30% of all respondents, the proportion was lower among 12- to 14-year-old boys. Given the comprehensive school system in the study country, computer-related ergonomic instructions should have been available to the entire study population. However, in 2001 when this study was carried out, health education had not yet been adopted by all schools as the separate subject it is today. Inclusion of ergonomic instructions for computer use in the school curriculum would ensure that all school-aged children receive basic ergonomic knowledge and skills.

Family was also reported by every third respondent as a source of computer-related ergonomic instructions. However, we do not know whether parents' own knowledge on computer ergonomics is appropriate, nor how intensively they control their children's computer habits. Adolescents seemed to be fairly active in finding out knowledge by experience: every eighth respondent reported being self-instructed, and the variety in the instruction methods was expressed in responses such as "self-instructed through experience", "I realized", or "I have trained myself". Self-instruction in computer ergonomics includes an element of doubt because of the risk that the self-sought information is less valid or can be misinterpreted by the adolescent.

The number of adolescents reporting ICT-related sources, e.g. television, radio and the internet, as a source of ergonomic instructions was surprisingly low (9%). Other studies have shown that adolescents often use the internet to find health information. In a study by Borzekowski and Rickert [23] on suburban tenth graders in the state of New York, about 50% had used the internet to obtain health information on diet, fitness, exercise etc. and considered such information valuable. Together with other media resources, the internet can also serve as an important tool for ergonomic information, because it provides easy access and a way of acquiring specific information that might otherwise be difficult to obtain.

Given the common occurrence of neck-shoulder and low back pain among adolescents, it is surprising that so few in our study, just 1%, had been trained in ergonomic issues by health care professionals. Usually, young people are willing to discuss a variety of health-related issues with school health care staff. Finland has comprehensive school health services with access to well-trained school health nurses. In a previous study from the United States, doctors, nurses or school nurses were frequently identified as the first persons consulted about health issues among fifth to twelfth grade students [24]. Computer-related ergonomics may have been a new subject for many doctors and nurses at the beginning of the 2000s, and unlike nutrition, exercise or growth, ergonomics was not a traditional issue for health counselling.

Overall, no relationship was found between ergonomic instructions and occurrence of health complaints, except that there were more eye-related complaints among adolescents who reported having received ergonomic instructions or being ergonomically self-instructed. Ergonomic instructions to arrange desks, chairs and screens in the right position or to take rest breaks were not statistically significantly associated with decreased levels of reported symptoms after adjusting for age, sex and computer use time. It is possible that adolescents with symptoms are more likely to seek instructions or remember receiving instructions. On the other hand, education does not necessarily result in actual changes in the workstation set-up. Our study cannot answer whether ergonomic instructions are effective for preventing computer-associated symptoms, but it showed that the adolescents who reported symptoms were not, in general, more informed than those who reported no symptoms.

Compared to earlier publications, the strength of this study was the large, nationwide sample size, depicting adolescents living in different conditions and locations. Furthermore, we examined several different health outcomes known to be related to adolescent computer use. Adolescents reported a wide selection of ergonomic sources to prevent computer-associated health complaints. The overall response rate was fairly good and the indirect analysis of non-respondents indicated no bias in the reported health complaints, computer use time or ergonomic instructions.

Some limitations of the study, however, require attention. This study was cross-sectional and causal inference on the relationship between computer time and computer-associated symptoms cannot be drawn from it. As the question used in the study measured respondents' perception of the causality of the relationship, it was a subjective measure: "Using a computer may cause health complaints (pains, aches, discomfort). Have you experienced any of these when using a computer?" Thus it was left to the respondents themselves to perceive whether any health complaints they may have are caused by specifically by computer use. Being a questionnaire survey, the rate of occurrence of symptoms and receiving instructions were based on self-reports, implying that memory bias is possible and differences between individuals' interpretations cannot be ruled out. Our questions on health complaints were measured with a simple three-point scale, including the alternatives "sometimes" and "often" which are not accurate measures of the frequency of symptoms. However, at group level, comparisons are valid. In our study, we used two separate questions to elicit the hours spent daily on a computer for e-mails, writing and information search, and the hours spent daily on playing digital games on computer, TV or console. Because it was difficult to distinguish between playing on computers versus console, the latter question was considered invalid and excluded from this study.

In the statistical analysis, we used logistic regression analysis to test the association between computer-associated health complaints and computer use time, adjusting for age, sex and parents' education. Other possible confounders, such as physical activity or school success, were not controlled. In the second analysis, we tested the associations between computer-associated health complaints and received ergonomic instructions. After first examining each health complaint in the model adjusted for age and sex, computer use time was included. This variable was considered a potential confounder and treated as a covariate, because it was a significant independent variable.

In this study, we explored a new aspect of computer-related adolescent lifestyle by asking where 12- to 18-year-old Finnish adolescents had received instructions in computer-related ergonomic issues and whether receiving instructions resulted in reduced symptoms. Further research is required to investigate the content and frequency of such instructions.

## Conclusions

Today's children and adolescents are the first generation to have grown up with information communication technology, and the use of computers in their everyday lives. With the increasing popularity of computers, the concern over potential computer-associated health complaints has increased. These complaints affect various anatomical sites, are common in this age group, increase with age, and increase with the longer time spent on a computer. This report shows that ergonomic instructions have so far failed to reach a substantial proportion of the Finnish school-age population, and furthermore that such instructions may not be associated with reduced levels of computer-associated symptoms. In addition, the reported sources of instructions vary greatly in terms of reliability.

## Competing interests

The authors declare that they have no competing interests.

## Authors' contributions

AHR initiated and designed the study, and LAS and RLK provided critical input in its all phases. PTH and AHR performed the main analysis, drafted the paper and co-ordinated subsequent revisions with the other authors. All authors read and approved the final manuscript.

## Pre-publication history

The pre-publication history for this paper can be accessed here:

http://www.biomedcentral.com/1471-2458/10/11/prepub
